# Unveiling the Psychological Impact of Climate Change: Generational Differences of Eco-Anxiety Among Italian Blood Donors

**DOI:** 10.1192/j.eurpsy.2025.612

**Published:** 2025-08-26

**Authors:** F. Marcolini, J. K. Rocholl, S. Tempia Valenta, D. De Ronchi, A. R. Atti

**Affiliations:** 1 Department of Biomedical and Neuromotor Sciences. University of Bologna, Bologna, Italy

## Abstract

**Introduction:**

Climate change is a pressing issue with significant impacts on both physical and mental health. Beyond threats to basic needs (e.g., water, clean air, food, and stable housing) and trauma from extreme weather events, its broader psychological effects, such as eco-anxiety, are gaining recognition. The American Psychological Association defines eco-anxiety as “a chronic fear of environmental doom,” which can lead to cognitive, emotional, and functional decline, along with somatic symptoms. Despite its growing recognition, climate change-related anxiety is still an emerging concept. In Italy, studies on eco-anxiety are limited but necessary, as the country is identified by the Intergovernmental Panel on Climate Change (IPCC) as particularly sensitive to climate change.

**Objectives:**

The aim of this study is to assess the prevalence of eco-anxiety in a population of Italian blood donors, with a particular focus on its distribution across different generational cohorts.

**Methods:**

An online questionnaire, structured on the Qualtrics platform, was administered via QR code among blood donors affiliated with AVIS (Associazione Volontari Italiani Sangue) in Bologna, Italy, in May 2024. Demographic and social data were collected, and the HEAS questionnaire was used to assess eco-anxiety.

**Results:**

The study included 1,795 participants (1,060 males, 727 females, 8 non-binary) with an average age of 46.6 years (range 18-70). The results revealed variation in the detection of eco-anxiety among participants (mean HEAS score: 5.09; SD: 5.84). Analyzing differences in the presence of eco-anxiety across various age groups, the mean HEAS score was found to be higher in the GenZ group, defined as those aged 18-28 years (mean: 8.65; SD: 7.50), compared to other groups. Kruskal-Wallis analyses confirmed statistically significant differences in the presence of eco-anxiety across generations (p-value < 0.001). The data indicate that, in the GenZ sample considered, the prevalence of eco-anxiety is 48.4%, representing nearly five out of ten young individuals (chi-square value: 81.3; p < 0.001). Spearman’s correlation and univariate logistic regression confirm the statistical validity of the association between generation and anxiety experiences related to climate change.

**Conclusions:**

The data highlight the alarming spread of eco-anxiety in contemporary society, particularly among younger generations. Given the significant consequences, it is crucial to deepen our understanding of this condition and its psychosocial determinants.

**Disclosure of Interest:**

None Declared

